# ASPECTS OF AMYLOID SCAN DISCLOSURE WHICH INFLUENCE PATIENT AND CARE PARTNER SATISFACTION DURING DEMENTIA DIAGNOSIS

**DOI:** 10.1093/geroni/igad104.2743

**Published:** 2023-12-21

**Authors:** Elyse Couch, Wenhan Zhang, Emmanuelle Belanger, Nicole DePasquale, Emily Gabois, Megan Shepherd-Banigan, Courtney Van Houtven, Terrie Wetle

**Affiliations:** Brown University, Providence, Rhode Island, United States; Duke University School of Medicine, Durham, North Carolina, United States; Brown University, Providence, Rhode Island, United States; Duke University School of Medicine, Durham, North Carolina, United States; Brown University School of Public Health, Providence, Rhode Island, United States; Duke University School of Medicine, Durham, North Carolina, United States; Duke University School of Medicine, Durham, North Carolina, United States; Brown University School of Public Health, Providence, Rhode Island, United States

## Abstract

Amyloid PET scans can be used to make a differential diagnosis of dementia or indicate a person’s risk of developing dementia in the future. The use of biomarkers for diagnosing dementia is not yet standard in clinical practice. Research is needed to better understand how to disclose the results from amyloid PET scans to patients and care partners. We interviewed 38 patients with MCI or dementia and 68 care partners from the CARE-IDEAS study to explore their experiences of receiving the results from amyloid PET scans as part of the diagnostic process for dementia. We were specifically interested in identifying aspects of amyloid disclosure which affected patient and care partner satisfaction. Patients had an average age of 73.5, were mostly male (65.8%), and Non-Hispanic White (67.6%). Care partners were mostly female (75.8%), caring for their spouse (87.1), with a mean age of 69.5. We identified 5 factors affecting patient and care partner satisfaction: 1) clarity of the explanation of the scan result and its implications, 2) discussion of potential options for treatment, research opportunities or follow-up support, 3) disclosure of scan results in person, 4) involvement of a care partner during disclosure, and 5) provision of a written summary of the scan result after disclosure. As diagnostic procedures for dementia evolve, it is important to consider their effect on patients and care partners. These findings can be used to inform the development of disclosure procedures for amyloid PET scans.

